# Surveillance of active human cytomegalovirus infection in hematopoietic stem cell transplantation (HLA sibling identical donor): search for optimal cutoff value by real-time PCR

**DOI:** 10.1186/1471-2334-10-147

**Published:** 2010-06-01

**Authors:** Renata MB Peres, Cláudia RC Costa, Paula D Andrade, Sandra HA Bonon, Dulcinéia M Albuquerque, Cristiane de Oliveira, Afonso C Vigorito, Francisco JP Aranha, Cármino A de Souza, Sandra CB Costa

**Affiliations:** 1 Department of Clinical Medicine, Faculty of Medical Sciences, University of Campinas - Unicamp, P.O.Box 6111, Zipe Code 13083-970, Campinas, SP, Brazil; 2 Bone Marrow Transplant Unit, Hemocenter, Faculty of Medical Sciences, University of Campinas - Unicamp, P.O.Box 6111, Zipe Code 13083-970, Campinas, SP, Brazil

## Abstract

**Background:**

Human cytomegalovirus (CMV) infection still causes significant morbidity and mortality after allogeneic hematopoietic stem cell transplantation (HSCT). Therefore, it is extremely important to diagnosis and monitor active CMV infection in HSCT patients, defining the CMV DNA levels of virus replication that warrant intervention with antiviral agents in order to accurately prevent CMV disease and further related complications.

**Methods:**

During the first 150 days after allogeneic HSTC, thirty patients were monitored weekly for active CMV infection by *pp65 *antigenemia, nested-PCR and real-time PCR assays. Receiver operating characteristic (ROC) plot analysis was performed to determine a threshold value of the CMV DNA load by real-time PCR.

**Results:**

Using ROC curves, the optimal cutoff value by real-time PCR was 418.4 copies/10^4 ^PBL (sensitivity, 71.4%; specificity, 89.7%). Twenty seven (90%) of the 30 analyzed patients had active CMV infection and two (6.7%) developed CMV disease. Eleven (40.7%) of these 27 patients had acute GVHD, 18 (66.7%) had opportunistic infection, 5 (18.5%) had chronic rejection and 11 (40.7%) died - one died of CMV disease associated with GVHD and bacterial infection.

**Conclusions:**

The low incidence of CMV disease in HSCT recipients in our study attests to the efficacy of CMV surveillance based on clinical routine assay. The quantification of CMV DNA load using real-time PCR appears to be applicable to the clinical practice and an optimal cutoff value for guiding timely preemptive therapy should be clinically validated in future studies.

## Background

Hematopoietic stem cell transplantation (HSCT) is an important therapeutic tool for treating malignant and non-malignant disorders, and the human cytomegalovirus (CMV) reactivation is common in these cases due to the immunocompromised state of patients [1].

Monitoring of its reactivation and preemptive or prophylactic treatment using ganciclovir are critical for HSCT recipients. However, because of the myelotoxicity of ganciclovir and its prolongation of periods of neutropenia, the prognosis in patients at low risk of developing CMV disease is not necessarily improved. Identification of patients at high risk of developing CMV is therefore believed to be important in management of HSCT recipients [2].

Despite major advances in prevention and preemptive treatment, CMV infection still causes significant morbidity and mortality following allogeneic hematopoietic stem cell transplantation (HSCT) [3] and occurs in 50-90% of allogeneic transplant recipients [4].

While detection of CMV *pp65 *antigenemia is still widely used for monitoring CMV infection and guide preemptive therapy in patients at risk of developing CMV disease [5,6], the quantification of CMV DNA in blood by PCR is emerging as an alternative to the *pp65 *antigenemia assay and may soon become the standard for the surveillance of CMV infection in allogeneic HSCT recipients [7], because it presents several advantages over the *pp65 *antigenemia assay, including an increased sensitivity for the detection of CMV reactivation, the reliable detection of CMV reactivation during severe neutropenia in the early post-transplant period, the shorter time required for the procedure, and the convenient processing of large numbers of specimens [8].

The aim of this study was to diagnosis and monitor active CMV infection in allogeneic HSCT patients. In addition, we have determined the proper cutoff level of CMV DNA load by real-time PCR for preemptive therapy, in order to switch the monitoring method from the *pp65 *antigenemia assay to real-time PCR method.

## Methods

### Patients

Between August 2006 and September 2008, a total of 558 consecutive blood samples were received by our Laboratory for the simultaneous determination of *pp65 *antigenemia, nested-PCR and real-time PCR in leukocytes. The samples came from 30 adult allogeneic HSCT recipients (17 males and 13 females; median age: 40.5, range: 16-56) with human leukocyte antigen (HLA) identical sibling donors at risk for CMV infection (CMV seropositive donor and/or recipient) at the Bone Marrow Transplant Unit, Hemocenter, University of Campinas - Unicamp, SP, Brazil (table 1). The median number of samples per patient was 20 (range: 5-23). These patients were prospectively monitored for active CMV infection at weekly intervals from D+0 to D+150 post-transplant. The protocol was designed in accordance with the requirements for research involving human subjects in Brazil, was approved by the Institutional Ethics Committee and an informed written consent was received from each patient.

**Table 1:** Demographic characteristics of the patients

Characteristic	
*Age - median in years (range)*	40.5 (16-56)
*Sex - male/female*	17/13
*Underlying disease - n (%)*	
Malignant disease	
Acute lymphocytic leukemia (ALL)	4 (13.3%)
Acute myelogenous leukemia (AML)	10 (33.3%)
Chronic lymphocytic leukemia (CLL)	1 (3.3%)
Chronic myelogenous leukemia (CML)	4 (13.3%)
Non-Hodgkin's lymphoma (NHD)	2 (6.7%)
Hodgkin's disease (HD)	3 (10%)
Multiple myeloma (MM)	1 (3.3%)
Myelofibrosis	2 (6.7%)
Non-malignant disease	
Severe aplastic anaemia (SAA)	3 (10%)
*Acute GVHD - (n%)*	11 (36.7%)
*Pretransplant CMV sorostatus - (n%)*	
D+/R+	30 (100%)
*Conditioning regimen - (n%)*	
Myeloablative transplant	
BU + FLU	7 (23.3%)
BU + Cy	6 (20%)
BU+ Cy + VP-16	3 (10%)
Cy + VP-16 + TBI	2 (6.7%)
Cy + TBI	1 (3.3%)
Non-myeloablative transplant	
FLU + TBI	9 (30%)
FLU + TBI + ARA-C	1 (3.3%)
*GVHD prophylaxis - (n%)*	
CsP	2 (6.7%)
CsP + MMF	7 (23.3%)
CsP + MTX	20 (66.7%)
CsP + MTX + Mitoxantrone + Cy	1 (3.4%)
*Stem cell source*	
Bone marrow	15 (50%)
Peripheral blood	15 (50%)
*Deaths*	13 (43.3%)

GVHD (Graft-versus-host disease); D (Donor); R (Recipient); BU (Bussulfan); FLU (Fludarabine); Cy (Cyclophosphamide); VP-16 (Etoposide); TBI (Total body irradiation); ARA-C (Cytarabine); CsP (Cyclosporine); MMF (Mycophenolate mofetil); MTX (Methotrexate).

### Management of CMV infection and disease

Active CMV infection, CMV recurrence and CMV disease were defined according to published recommendations [9].

Active CMV infection is defined as isolation of CMV virus or detection of viral proteins or nucleic acid from any body fluid or tissue specimen. CMV recurrence is defined as a new detection of CMV infection in a patient previously diagnosed with infection and in whom virus was not detected for an interval of at least four weeks during active surveillance. CMV disease, which was defined by immunohistochemical analysis of biopsy specimens, is followed by clinical signs and symptoms, such as: unexplained fever (> 38°C), leukopenia (white blood cells < 3.5 × 10^9^/L) and/or thrombocytopenia (platelet count < 100 × 10^9^/L), gastrointestinal symptoms, arthralgia, hepatitis, enteritis, retinitis, pneumonitis, colitis, oesophagitis, encephalitis [10]. Probable CMV disease is defined when clinical signs and symptoms are present, but without accomplishment of biopsy.

### Therapy

Preemptive therapy was used to prevent CMV disease and was initiated upon a positive *pp65 *cell/3 × 10^5 ^PML result ≥1 and/or two or more consecutive positive nested-PCR results. Active CMV infection was treated with GCV (5 mg/Kg twice daily, i.v) for seven days, followed by a maintenance dose of 5 mg/Kg/day, i.v., three times a week for four weeks, and CMV disease was treated with GCV (5 mg/Kg twice daily, i.v) for 21 days, followed by a maintenance dose of 5 mg/Kg/day, i.v., three times a week for four weeks.

### Specimen processing

A 12 mL of EDTA-treated blood was collected from each patient. Four mL were used for CMV *pp65 *antigenemia assay and processed within 6 hours. Eight mL were used for nucleic acid extraction by manual phenol-chloroform method.

### CMV pp65 antigenemia assay

Antigenemia is based on immunocytochemical detection of the early structural, lower matrix protein (*pp65*) in polymorphonuclear leukocytes (PML), as described by Van der Bij *et al*. [10], with some modifications, as described by Bonon *et al*. [11]. Blood samples were collected in EDTA-containing tubes and transferred to the laboratory within 6 hours. Leukocytes were isolated by dextran sedimentation method, followed by erythrocyte lysis. The cell pellet was suspended in phosphate-buffered saline (PBS), and the polymorphonuclear leukocytes (PML) were then centrifuged to prepare cytospin slides (3 × 10^5 ^PML per slide). The slides were air-dried and fixed in formaldehyde, then immunostained with monoclonal antibodies (Iq Products, Netherlands), and reacted with peroxidase-labeled anti-mouse conjugate (HRP, Biotest, Dreieich, Germany). The test was carried out in duplicate. Results were expressed as number of positive cells per 3 × 10^5 ^PML.

### Nucleic acid extraction by manual phenol-chloroform method

CMV DNA was extracted from peripheral blood collected in EDTA-containing tubes. Erythrocytes and leukocytes were lysed. The sample was transferred to a tube containing 400 μl of extraction buffer (Tris-HCl [10 mM, pH 7.6], KCl [10 mM], MgCl^2 ^[10 mM], NaCl [0.4 M], EDTA [2 mM]) and 25 μl of sodium dodecyl sulfate [10%], and incubated at 55°C for 30 minutes. The supernatant was then purified by phenol-chloroform isoamilic alcohol (24:1), followed by purification with chloroform isoamilic alcohol (24:1). DNA was precipitated with ethanol, resuspended in 25 μl of distilled water and stored at -20°C until use. These extracted DNA samples were used for both qualitative (nested-PCR) and quantitative PCR (real-time PCR) assays.

### Nested-PCR

CMV DNA in blood specimens was detected by nested-PCR using primers, as described by Demmler *et al*. [12] and Shibata *et al*. [13]. CMV DNA was extracted from peripheral blood by a manual phenol-chloroform method. The primers were selected from the MIE region of CMV AD169. The size of PCR amplification products was 159 base pairs. The same protocol was used to amplify the human β-globin gene sequence to guarantee the quality of the extracted DNA. All experiments had two negative controls (one without DNA and the other with a human PBL DNA preparation known to be negative for CMV DNA) and one positive control (by CMV strain AD169 aliquot).

### Real-time PCR

The PCR primers and probe sequences were selected from the US17 region of CMV strain AD169. The forward and reverse CMV primers were 5' GAAGGTGCAGGTGCCCTG 3' and 5' GTGTCGACGAACGACGTACG 3', respectively. The TaqMan^® ^probe selected between both primers was fluorescence labeled with 6-carboxyfluorescein at the 5' end as the reporter dye and 6-carboxytetramethylrhodamine at the 3' end as the quencher (5' FAM ACGGTGCTGTAGACCCGCATACAAA TAMRA3'). A search of databases indicated that neither the primers nor the probe shared significant homology with any known nucleotide sequence. The oligonucleotide synthesis was provided by IDT^®^, Inc (Integrated DNA Technologies). The reference standard curve for calibration of CMV copy numbers was constructed inserting the US17 amplicon, respectively, into a plasmid *PROMEGA P GEM - T Easy Vector System I *(Promega), using a cloning strategy, and propagated in competent *Escherichia coli *cells. For this construct, plasmid DNA was purified on columns with SV Wizard Purification System (Promega), DNA concentration was determined by measuring OD260, using a NanoDrop ND-1000 spectrophotometer and the corresponding copy number was then calculated. The construct was serially diluted in water within a range of 10^2 ^to 10^7 ^copies/μl. The real-time PCR was performed with a 12 μl mixture containing: 3 mM MgCl_2_; 10 μM dATP, dCTP, dGTP, dTTP; 5 U/μl of Platinum Taq (Invitogen), 60 ng DNA templates, 150 nM of the forward and reverse primers (CMVUS17F-CMVUS17R for CMV detection) and 2 μM of the specific TaqMan^® ^CMV probe (PE Applied Biosystems). The single PCR was performed in 96-well microliter plates under the following conditions: 1 cycle at 50°C for 2 minutes, 95°C for 10 minutes and 45 cycles at 95°C for 15 seconds and 60°C for 1 minute. The β-actin gene amplification was performed under the same PCR conditions described above for the reaction control using 2 μM β-actin probe (FAM™ Probe), 3 μM β-actin forward primer, and 3 μM β-actin reverse primer (TaqMan^® ^β-actin detection reagents - Applied Biosystems).

### Statistical analysis

Receiver operating characteristic (ROC) plot analysis was performed to determine a threshold value of the CMV DNA load in peripheral blood leukocytes (PBL) for initiating preemptive treatment. Using 1 *pp65 *antigen positive cell/3 × 10^5 ^PML as the reference standard, the best sensitivity and specificity were calculated to determine optimal cutoff value for CMV DNA copies. Probability density function was used to describe the probability of occurrence of active CMV infection during the period of monitoring. A contingency table analysis was used to calculate the sensitivity, specificity and positive (PPV) and negative predictive values (NPV) of nested-PCR and real-time PCR using *pp65 *antigenemia ≥ 1 positive cell as the reference standard. The Fisher's exact test was used to calculate the probability of association among active CMV infection and CMV disease, acute graft-versus host disease (GVHD), opportunist infection, graft rejection and death. Statistical package that has been used is R Development Core Team (2009).

## Results

### Active CMV infection

**Figure 1:**
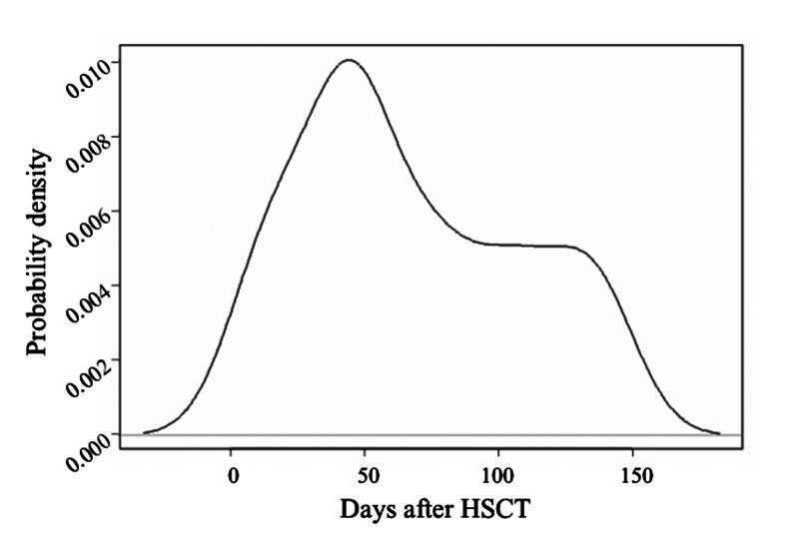
**Probability density of active CMV infection**. Seasonal variation of active CMV infection over a 150 days after HSCT period. The highest incidence occurred during the second post-transplant month with maximum value of probability density of 0.010 at day 44.4 after HSCT

In this study, we considered active CMV infection detection one or more positive cells by *pp65 *antigenemia assay and/or two or more consecutive positive nested-PCR and/or load CMV ≥ 418.4 copies/10^4 ^peripheral blood leukocytes (PBL) by real-time PCR. The highest incidence of active CMV infections occurred during the second post-transplant month (31 - 60 days) with a percentage of active CMV infection of 76.7% and maximum value of probability density of 0.01 at day 44.4 after HSCT (figure 1). Twenty seven (90%) of the 30 analyzed patients had active CMV infection, nine (30%) had recurrence of CMV infection, two developed probable CMV disease (6.7%) and two (6.7%) developed CMV disease. Twenty one (77.8%) of the 27 patients who had active CMV infection received preemptive antiviral therapy, 11 (40.7%) had occurrence of acute GVHD, 18 (66.7%) had opportunist infection, five (18.5%) had chronic rejection and 11 (40.7%) died (table 2).

**Table 2:** Occurrence of active CMV infection *versus *complications associated with HSCT

Active CMV Infection					
	**Positive**	**Negative**	**Median (days)**	**Range**	p*
				
Recurrence of Active CMV Infection	9/27	0/3	114	46 - 152	NS
CMV Disease	2/27	0/3	86.5	80 - 93	NS
Acute GVHD	11/27	0/3	77	26 - 96	NS
Opportunist Infection	18/27	3/3	20	3 - 348	NS
Graft Rejection	5/27	0/0	166	51 - 199	NS
Death	11/27	2/3	203	33 - 534	NS

CMV, human cytomegalovirus; GVHD, graft-versus host disease; NS, not significant; *Fisher's exact test.

### Incidence and outcome of CMV disease

Two of the 30 patients developed CMV disease after a median of 86.5 days (range 80-93 days) from HSCT. Both had gastrointestinal disease with manifestation of CMV infection in gastrointestinal tract biopsy specimens, and one of them died due to CMV disease associated with GVHD and bacterial infection at 252 days after HSCT.

Two patients had probable CMV disease after a median of 70.5 days (range 30-111 days) from HSCT, one of them had gastrointestinal infection and the other one had interstitial pneumonia, both without accomplishment of biopsy. The patient who had interstitial pneumonia died of this cause at 33 days after HSCT.

### Comparisons of the three assays

**Figure 2:**
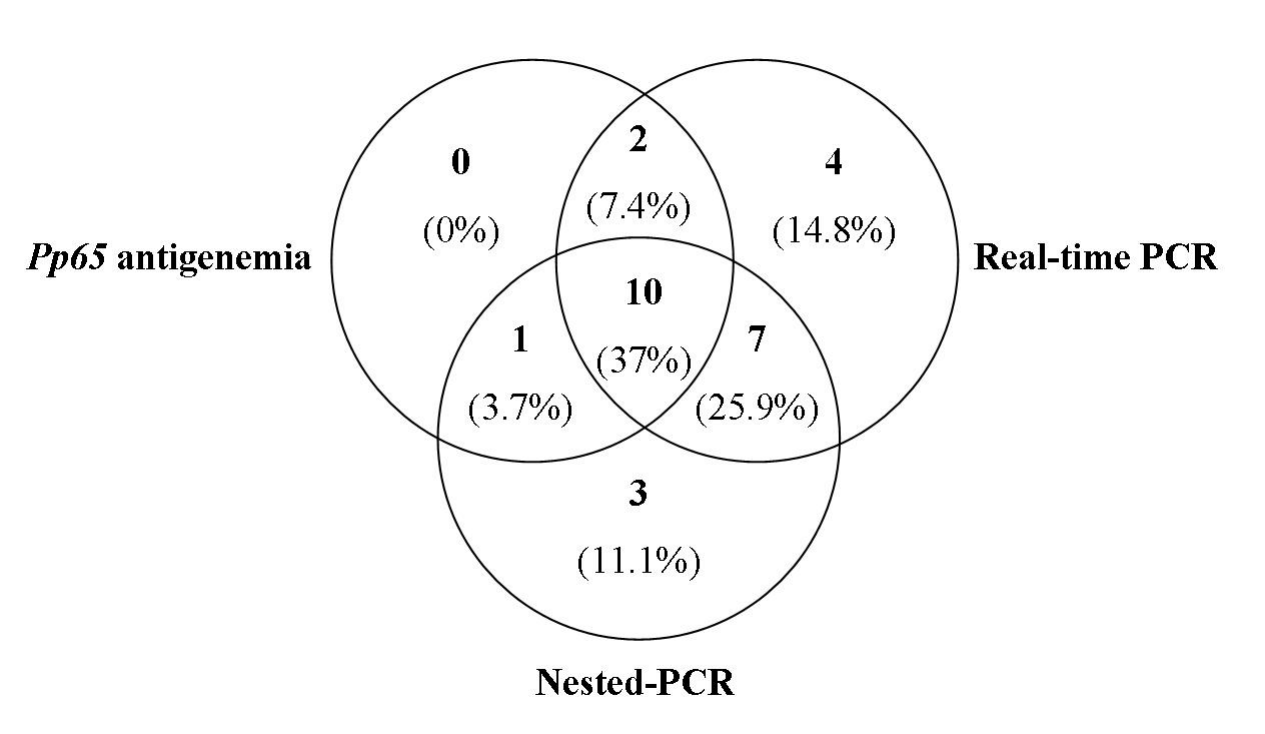
**Active CMV infection stratification by diagnostic test**. Absolute and relative number of patients with positive CMV samples stratified for diagnostic assays (n = 27). Active CMV infection: ≥ 1 cell *pp65 *positive/3 × 10^5 ^PML, and/or 2 or more consecutive positive nested-PCR and/or load CMV ≥ 418.4 copies/10^4 ^PBL by real-time PCR. All tests were performed weekly from aliquots of the same blood sample

A total of 558 weekly samples were obtained from 30 patients and analyzed by *pp65 *antigenemia, nested-PCR and real-time PCR assays. A total of 35 samples obtained from 13 patients were positive by *pp65 *antigenemia, 154 samples from 21 patients were positive by nested-PCR and 78 samples from 23 patients were positive by real-time (using the cutoff value of 418.4 copies/10^4 ^PBL). Ten patients were positive by the three assays (*pp65 *antigenemia, nested-PCR and real-time PCR), two patients were positive by *pp65 *antigenemia and real-time PCR, one patient was positive by *pp65 *antigenemia and nested-PCR, seven patients were positive by nested-PCR and real-time PCR, four patients were positive by real-time PCR only, and three patients were positive by nested-PCR only (figure 2). Using *pp65 *antigenemia as the reference standard, the sensitivity and specificity of nested-PCR were 84.6% and 41.2% and the positive predictive value (PPV) and the negative predictive value (NPV) were 52.4% and 77.8%, respectively. The real-time PCR showed a sensitivity of 92.3%, specificity of 35.3%, PPV of 52.2% and NPV of 85.7%, using the cutoff value of 418.4 copies/10^4 ^PBL (table 3).

**Table 3:** Results of contingency table analysis using *pp65 *antigenemia as a reference standard

	**Nested-PCR**	**Real-time PCR**
	
Sensitivity	84.6%	92.3%
Specificity	41.2%	35.3%
PPV	52.4%	52.2%
NPV	77.8%	85.7%

PPV, positive predictive value; NPV, negative predictive value.

### Time to detection of active CMV infection by pp65 antigenemia, nested-PCR and real-time PCR in transplanted patients

Thirteen (43.3%) of the 30 patients monitored developed positive *pp65 *antigenemia after a median of 40 days (range 29-152 days), 21 (70%), developed a consecutive positive nested-PCR after a median of 33 days (range 0-126 days), while 23 (76.7%) developed positive real-time PCR after a median of 40 days (range 0 - 119 days) from HSCT (table 4).

**Table 4:** Time to until detection of active CMV infection by *pp65 *antigenemia, nested-PCR and real-time PCR

	N° Patients	Median (days after HSCT)	Range
Patients with active CMV infection	27 (90%)	33	0 - 119
Positive *pp65 *antigenemia (%)	13 (43.3%)	40	29 - 152
Positive nested-PCR (%)	21 (70%)	33	0 - 126
Positive real-time PCR (%)	23 (76.7%)	40	0 - 119

CMV, human cytomegalovirus.

### Determination of CMV DNA cutoff value for real-time PCR

**Figure 3:**
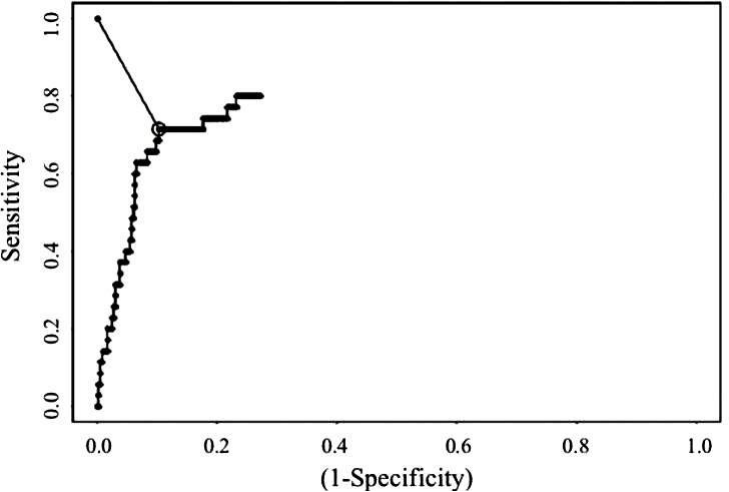
**ROC curve to determine optimal cutoff value by real-time PCR**. ROC curve graphing sensitivity *versus *(1-specificity) for the prediction of determination of active CMV infection using *pp65 *antigenemia (1 positive *pp65 *cells/3 × 10^5 ^PML) as the reference standard for establishing the optimal cutoff level for real-time PCR. The optimal cutoff value for real-time PCR in peripheral blood leukocytes was 418.4 copies/10^4 ^PBL (sensitivity 71.4%; specificity 89.7%)

Using ROC curves, optimal cutoff points for viral load by real-time PCR in peripheral blood leukocytes (PBL) were calculated for one specific *pp65 *antigenemia value: ≥1 positive cell/3 × 10^5 ^PML. The optimal cutoff value for real-time PCR in peripheral blood leukocytes was 418.4 copies/10^4 ^PBL (sensitivity 71.4%; specificity 89.7%) (Figure 3).

## Discussion

We have established a new method of diagnosis of CMV infection using real-time PCR based on TaqMan^® ^technology. Several studies, such as Machida *et al. *[2], reported that PCR is a very useful method for early diagnosis of CMV disease, but it may be frequently too sensitive for clinical use and do not necessarily indicate an imminent risk of developing CMV disease. However, we overcame this problem using receiver operating characteristic (ROC) plot analysis to determine a threshold value of the CMV DNA load for initiating treatment. The sensitivities and specificities for US17 were calculated to determine optimal cutoff value for CMV DNA copies.

In this study, we used three different detection assays (*pp65 *antigenemia, nested-PCR and real-time PCR) for monitoring active CMV infection in allogeneic stem cell transplant recipients. We could observe a high incidence of active CMV infection occuring during the second post-transplant month (31 - 60 days), with a maximum value of probability density 44.4 days after HSCT, corroborating data in the literature, which reports that the highest incidence of CMV infections occur during the first three post-transplant months [14], secondary to the greatest degree of immunosuppression [15,16].

The incidence of active CMV infection was high (27 of 30 patients, 90%) after a median of 33 days (range 0-119 days), and only two (6.7%) patients had CMV disease, both in the gastrointestinal tract. Only one (3.3%) of 30 patients monitored in this study died of CMV disease, in agreement with the low rates of CMV-related mortality cited in published reports [17,18].

The incidence of active CMV infection was comparable to that found in previous reports on HSCT. Bonon *et al*. [11] noted active CMV infection in 55 (79.7%) of 69 analyzed patients, considering that six (8.7%) developed CMV disease. Gentile *et al*. [19] evaluated 38 HSCT recipients. Among them, 25 (70%) had active CMV infection and three (7.9%) were pathologically diagnosed with CMV colitis.

The high incidence of active CMV infection detected in our patients is associated to no prophylactic treatment and pretransplant CMV sorostatus of donors and recipients (D+/R+:100%). This group is identified as high-risk patients to develop active CMV infection and CMV disease [20].

In this study, the absence of statistical significance among active CMV infection and recurrence of CMV infection, CMV disease, GVHD, opportunist infection, chronic rejection and death may be influenced by the small number of patients, the preexisting heterogeneity of conditioning regimens, GVHD prophylaxis and hematological diseases, according to Gentile *et al*. [19].

Several studies have attempted to determine a CMV DNA copy number equivalent to the levels of *pp65 *antigenemia and to implement CMV PCR as *pp65 *antigenemia in clinical practice [21]. Identification of a cutoff level for real-time PCR assay would be an important indicator of time to initiate an anti-CMV treatment [20], would reduce the number of patients treated with preemptive therapy who are not destined to develop CMV disease [6] and the duration of preemptive therapy might be shortened by real-time PCR guided manner, because more rapid negative conversion of viral reactivation was detected using this assay [8].

Choi *et al*. [8] suggested cutoff values of approximately 3 × 10^4 ^copies/mL in low-risk patients and of approximately 2 × 10^4 ^copies/mL in high-risk patients. Cariani *et al*. [22] defined a positive cutoff value equivalent to 9960 copies/mL in HSCT. Garrigue *et al*. [23] reported that thresholds of 10 and 50 positive cells/2 × 10^5 ^cells were equivalent to 3.3 log10 copies/mL (2000 copies/mL) and 3.8 log10 copies/mL (6300 copies/mL), respectively. Kalpoe *et al*. [24] suggested that preemptive therapy could be initiated at CMV DNA load of 1000 copies/mL and then increased by 10-fold per week at the first episode of CMV reactivation. Lilleri *et al*. [25] suggested a cutoff value of 10000 copies/mL for initiating preemptive therapy in HSCT recipients, and Graffari *et al*. [21] previously defined a positive cutoff value of greater than 1000 copies/2 × 10^5 ^PBL in HSCT recipients.

In our study, we have calculated the optimal cutoff viral load using ROC curve for a *pp65 *antigenemia value ≥1 positive cell. The analysis of data indicated that a CMV DNA level of 418.4 copies/10^4 ^PBL was a convenient value for discriminating between latent infections and those requiring preemptive therapy. This specific *pp65 *antigenemia value has been used in our Centre as the threshold for initiating preemptive therapy in HSCT recipients with an incidence of CMV disease lower than 7%.

It is difficult to compare data from different centers because real-time PCR methods are not well standardized and use different target sequences, primer sets, and extraction and detection methods, which result in different analytical performances [7].

Given the lack of standardization of CMV monitoring by real-time PCR, the cutoff definition for predicting CMV disease and initiating preemptive antiviral therapy remain a question [26]. The results shows that studies evaluating this assay are highly heterogeneous, there is no available international standard for CMV PCR, and each study determined its own assays characteristics for their own setup [23].

Most reports on detecting CMV DNA by real-time PCR have used 'in-house' assays with variable primers for the same gene or different genes (e.g. the immediate early CMV gene or the CMV DNA polymerase gene), making it difficult to extrapolate results from one institution to another [21,27]. Assay sensitivity varies greatly depending on the methodology used. Commercial diagnostic tests could avoid variability between home-brewed assays, improving reproducibility and standardization of the results [28].

Further studies to correct current cutoff value and to validate the optimal cutoff value for the initiation of preemptive therapy are currently underway at our HSCT center.

Conversely, we thought that high negative predictive value (85.7%) could allow us to avoid unnecessary preemptive therapy with a fair aliquot of patients. The optimal cutoff value by real-time PCR for therapeutic intervention needs to be clearly defined to determine the maximal specificity for CMV disease, and to define its value for prognosis and use in therapeutic clinical trials.

## Conclusion

The low incidence of CMV disease in HSCT recipients in our study attests to the efficacy of CMV surveillance, based on the *pp65 *antigenemia assay and nested-PCR (assay used in clinical routine). The quantification of CMV DNA load using real-time PCR appears to be applicable to the clinical practice and an optimal cutoff value for guiding timely preemptive therapy should be clinically validated in future studies. For this reason, we think that real-time PCR can be used complementarily to *pp65 *antigenemia screening to monitor preemptive therapy for presenting high sensitivity, in addition to be an alternative for CMV diagnoses in cases of samples collected before engraftment due to the lack of leukocytes during the period of aplasia, in neutropenic patients and in occurrence of CMV gastrointestinal disease.

## List of Abbreviations

CMV: human cytomegalovirus; HSCT: hematopoietic stem cell transplantation; ROC: receiver operating characteristic; PBL: peripheral blood leukocytes; GVHD: graft-versus host disease; GCV: ganciclovir; PCR: polymerase chain reaction; HLA: human leukocyte antigen; PML: polymorphonuclear leukocytes; EDTA: ethylenediamine tetraacetic acid; PBS: phosphate-buffered saline; PPV: positive predictive value; NPV: negative predictive value

## Competing interests

The authors declare that they have no competing interests.

## Authors' contributions

All the authors contributed substantially to the study. RMBP and CRCC designed the study, contributed to data analysis, carried out the immunoassays and wrote the manuscript. PDA and SHAB contributed to data analysis and drafted the manuscript. DMA and CO carried out the immunoassays. ACV, FJPA and CAS coordinated and participated in the study. SCBC contributed to the study design, conducted and coordinated the laboratory studies and wrote the manuscript. All authors read and approved the final manuscript.

## Pre-publication history

The pre-publication history for this paper can be accessed here:

http://www.biomedcentral.com/1471-2334/10/147/prepub

## References

[B1] Nichols WG Corey L Gooley T Davis C Boeckh M High risk of death due to bacterial and fungal infection among cytomegalovirus (CMV)-seronegative recipients of stem cell transplants from seropositive donors: evidence for indirect effects of primary CMV infection J Infect Dis 2002 185 273 282 10.1086/338624 11807708

[B2] Machida U Kami M Fukui T Kazuyama Y Kinoshita M Tanaka Kanda Y Ogawa S Honda H Chiba S Mitani K Muto Y Osumi K Kimura S Hirai H Real-Time automated PCR for early diagnosis and monitoring of cytomegalovirus infection after bone marrow transplantation J Clin Microbiol 2000 38 2536 2542 10878039 10.1128/jcm.38.7.2536-2542.2000 PMC86962

[B3] Boeckh M Leisenring W Riddell SR Bowden RA Huang ML Myerson D Stevens-Ayers T Flowers ME Cunningham T Corey L Late cytomegalovirus disease and mortality in recipients of allogeneic hematopoietic stem cell transplants: importance of viral load and T-cell immunity Blood 2003 101 407 414 10.1182/blood-2002-03-0993 12393659

[B4] Laso JF Diagnostico Diferencial en Medicina Interna 2005 2 Barcelona: Elsevier Espanã 497

[B5] Patel R Paya CV Infections in solid organ transplant recipients Clin Microbiol Rev 1997 10 1 86 124 8993860 10.1128/cmr.10.1.86 PMC172945

[B6] Boeckh M Boivin G Quantification of Cytomegalovirus: methodologic aspects and clinical applications Clin Microbiol Rev 1998 11 5333 5354 10.1128/cmr.11.3.533 PMC88895 9665982

[B7] Gimeno C Solano C Latorre JC Hernández-Boluda JC Clari MA Remigia MJ Furió S Calabuig M Tormo N Navarro D Quantification of DNA in Plasma by an Automated Real-Time PCR Assay (Cytomegalovirus PCR Kit) for Surveillance of Active Cytomegalovirus Infection and Guidance of Preemptive Therapy for Allogeneic Hematopoietic Stem Cell Transplant Recipients J Clin Microb 2008 46 10 3311 3318 10.1128/JCM.00797-08 PMC2566127 18753357

[B8] Choi SM Lee DG Lim J Park SH Choi JH Yoo JH Lee JW Kim Y Han K Min WS Shin WS Kim CC Comparison of Quantitative Cytomegalovirus Real-time PCR in Whole Blood and pp65 Antigenemia Assay: Clinical Utility of CMV Real-time PCR in Hematopoietic Stem Cell Transplant Recipients J Korean Med Sci 2009 24 4 571 578 10.3346/jkms.2009.24.4.571 19654935 PMC2719194

[B9] Ljungman P Griffiths P Paya C Definitions of cytomegalovirus infection and disease in transplant recipients Clin Infect Dis 2002 34 1094 1097 10.1086/339329 11914998

[B10] van der Bij W Schirm J Torensma R van Son WJ Tegzess AM The TH Comparison between viremia and antigenemia for detection of cytomegalovirus in blood J Clin Microbiol 1988 26 12 2531 2535 2852670 10.1128/jcm.26.12.2531-2535.1988 PMC266939

[B11] Bonon SHA Menoni SM Rossi CL De Souza CA Vigorito AC Costa DB Costa SCB Surveillance of cytomegalovirus infection in haematopoietic stem cell transplantation patients J Infect 2005 50 130 137 10.1016/j.jinf.2003.11.010 15667914

[B12] Demmler GJ Buffone GJ Schimbor CM May RA Detection of cytomegalovirus in urine from newborns by using polymerase chain reaction DNA amplification J Infect Dis 1988 158 1177 1184 2848897 10.1093/infdis/158.6.1177

[B13] Shibata D Martin WJ Appleman MD Causey DM Leedom JM Arnheim N Detection of cytomegalovirus DNA in peripheral blood of patients infected with human immunodeficiency virus J Infect Dis 1988 158 1185 1192 2848898 10.1093/infdis/158.6.1185

[B14] Schroeder R Michelon T Fagundes I Bortolotto A Lammerhirt E Oliveira J Santos A Bittar A Keitel E Garcia V Neumann J Saitovitch D Cytomegalovirus disease latent and active infection rates during the first trimester after kidney transplantation Transplant Proc 2004 36 896 898 10.1016/j.transproceed.2004.03.085 15194308

[B15] Opelz G Döhler B Ruhenstroth A Cytomegalovirus prophylaxis and graft outcome in solid organ transplantation: a collaborative transplant study report Am J Transplant 2004 4 928 936 10.1111/j.1600-6143.2004.00451.x 15147427

[B16] Sagedal S Nordal KP Hartmann A Sund S Scott H Degré M Foss A Leivestad T Osnes K Fauchald P Rollag H The impact of cytomegalovirus infection and disease on rejection episodes in renal allograft recipients Am J Transplant 2002 2 850 856 10.1034/j.1600-6143.2002.20907.x 12392291

[B17] Machado CM Dulley FL Boas LS Castelli JB Macedo MC Silva RL Pallota R Saboya RS Pannuti CS CMV pneumonia in allogenic BMT recipients undergoing early treatment or pre-emptive ganciclovir therapy Bone Marrow Transplant 2000 26 413 417 10.1038/sj.bmt.1702526 10982288

[B18] Martino R Caballero MD Canals C San Miguel J Sierra J Rovira M Solano C Bargay J Pérez-Simon J León A Sarrá J Brunet S de la Cámara R alloPBSCT and Infectious/nonifectious Complications Subcommittees of the Grupo Español de Transplante Hematopoyético (GETH) Reduced-intensity conditioning reduces the risk of severe infections after allogenic peripheral blood stem cell transplantation Bone Marrow Transplant 2001 28 341 347 10.1038/sj.bmt.1703150 11571505

[B19] Gentile G Picardi A Capobianchi A Spagnoli A Cudillo L Dentamaro T Tendas A Cupelli L Ciotti M Volpi A Amadori S Martino P de Fabritiis P A prospective study comparing quantitative Cytomegalovirus (CMV) polymerase chain reaction in plasma and pp65 antigenemia assay in monitoring patients after allogeneic stem cell transplantation BMC Infect Dis 2006 6 167 10.1186/1471-2334-6-167 17118205 PMC1664570

[B20] Szczepura A Westmoreland D Vinogradova Y Fox J Clark M Evaluation of molecular techniques in prediction and diagnosis of cytomegalovirus disease in immunocompromised patients Health Technology Assessment 2006 10 10 175 10.3310/hta10100 16595079

[B21] Ghaffari SH Obeidi N Dehghan M Alimoghaddam K Gharehbaghian A Ghavamzadeh A Monitoring of Cytomegalovirus Reactivation in Bone Marrow Transplant Recipients by Real-time PCR Pathol Oncol Res 2008 14 4 399 409 10.1007/s12253-008-9030-3 18392952

[B22] Cariani E Pollara C Valloncini B Perandin F Bonfanti C Manca N Relationship between pp65 antigenemia levels and real-time quantitative DNA PCR for Human Citomegalovirus (HCMV) management in immunocompromised patients BMC Infect Dis 2007 7 138 10.1186/1471-2334-7-138 18036216 PMC2222614

[B23] Garrigue I Boucher S Couzi L Caumont A Dromer C Neau-Cransac M Tabrizi R Schrive MH Fleury H Lafon ME Whole blood real-time quantitative PCR for cytomegalovirus infection follow-up in transplant recipients J Clin Virol 2006 36 72 75 10.1016/j.jcv.2006.01.002 16481215

[B24] Kalpoe JS Kroes AC de Jong MD Schinkel J de Brouwer CS Beersma MF Claas EC Validation of clinical application of cytomegalovirus plasma DNA load measurement and definition of treatment criteria by analysis of correlation to antigen detection J Clin Microbiol 2004 42 1498 1504 10.1128/JCM.42.4.1498-1504.2004 15070995 PMC387533

[B25] Lilleri D Gerna G Furione M Bernardo ME Giorgiani G Telli S Baldanti F Locatelli F Use a DNAemia cut-off for monitoring human cytomegalovirus infection reduce the number of preemptively treated children and young adults receiving heamatopoietic stem cell transplantation as compared to qualitative pp65 antigenemia Blood 2007 110 2757 2760 10.1182/blood-2007-03-080820 17579182

[B26] Deback C Fillet AM Dhedin N Barrou B Varnous S Najioullah F Bricaire F Agut H Monitoring of human cytomegalovirus infection in immunosuppressed patients using real-time PCR on whole blood J Clin Virol 2007 40 173 179 10.1016/j.jcv.2007.08.014 17904901

[B27] Ruell J Barnes C Mutton K Foulkes B Chang J Cavet J Guiver M Menasce L Dougal M Chopra R Active CMV disease does not always correlate with viral load detection Bone Marrow Transplant 2007 40 55 61 10.1038/sj.bmt.1705671 17468776

[B28] Mart'ın-D'avila P Fort'un J Guti'errez C Mart'ı-Belda P Candelas A Honrubia A Barcena R Mart'ınez A Puente A de Vicente E Moreno S Analysis of a quantitative PCR assay for CMV infection in liver transplant recipients: an intent to find the optimal cut-off value J Clin Virol 2005 33 138 144 10.1016/j.jcv.2004.09.032 15911429

